# Predicting the Absolute Risk of Ischemic Stroke in Asian Patients with Atrial Fibrillation: Comparing the COOL-AF Risk Score with CARS/mCARS Models for Absolute Risk and the CHA2DS2-VASc Score

**DOI:** 10.3390/jcm12072449

**Published:** 2023-03-23

**Authors:** Rungroj Krittayaphong, Arjbordin Winijkul, Poom Sairat, Gregory Y. H. Lip

**Affiliations:** 1 Division of Cardiology, Department of Medicine, Faculty of Medicine Siriraj Hospital, Mahidol University, 2 Wanglang Road, Bangkoknoi, Bangkok 10700, Thailand; 2 Liverpool Centre for Cardiovascular Science at University of Liverpool, Liverpool John Moores University and Liverpool Heart & Chest Hospital, Liverpool L14 3PE, UK; 3 Department of Clinical Medicine, Aalborg University, 9220 Aalborg, Denmark

**Keywords:** atrial fibrillation, prediction model, CARS, ischemic stroke

## Abstract

Background: The aims of this study were (1) to validate the CARS and mCARS methods in an Asian population with atrial fibrillation (AF) and (2) to compare the CARS and mCARS models for absolute risk using the COOL-AF method and CHA_2_DS_2_VASc scores for the prediction of ischemic stroke or systemic embolism (SSE). Methods: We analyzed the results from a prospective nationwide multicenter AF registry. Follow-up data were collected for 3 years. The main outcomes were SSE. Predictive models of the 3-year SSE of the COOL-AF model, the CHA_2_DS_2_VASc score, the CARS for the no-OAC group, and the mCARS for the OAC group were developed and evaluated by C-statistics, and calibration plots were created for the whole group, as well as for oral anticoagulant (OAC) users and no-OAC patients. Results: We studied 3405 patients (mean age: 67.8 years; 58.2% male, 75.4% OAC). The incidence rates of SSE were 1.51 (1.26–1.78), 1.93 (1.39–2.60), and 1.37 (1.10–1.68) for all patients, no-OAC patients, and OAC patients, respectively. For the whole population, the COOL-AF score had a C-statistic of 0.697 (0.682–0.713), which was superior to the CHA_2_DS_2_-VASc [0.655 (0.639–0.671)]. For the no-OAC group, the CARS predicted SSE with a C-statistic of 0.685 (0.652–0.716), which was similar to the CHA_2_DS_2_-VASc [0.684 (0.651–0.7150] and COOL-AF models [0.692 (0.659–0.723)]. For the OAC group, the mCARS had a C-statistic of 0.687 (0.669–0.705) that was similar to the COOL-AF [0.704 (0.686–0.721)] and better than the CHA_2_DS_2_-VASc score [0.655 (0.637–0.674)]. Conclusions: The calculation of the individual absolute risks using the CARS and mCARS models can predict SSE in an Asian population. Small differences were evident between the COOL-AF and CHA_2_DS_2_-VASc scores.

## 1. Introduction

Non-valvular atrial fibrillation (AF) can lead to an increased risk of ischemic stroke or systemic embolism (SSE) that is approximately five-fold that of the general population [1]. Guideline recommendations for the assessment of the risk of SSE are currently based on simple clinical risk scores, such as the CHA_2_DS_2_VASc score [2,3,4,5]. Given the limitations of clinical risk scores such as the CHA_2_DS_2_VASc, the default is to offer stroke prevention to all patients with AF unless they are identified as being at a very low risk [2,3,4,5,6]. However, since OACs can increase the risk of major bleeding, physicians should balance the benefits and risks of OACs for each individual patient, and shared decision making with patients is recommended [7,8]. Despite the evidence that use of OACs can reduce the risk of ischemic stroke in patients with AF, there are residual risks of ischemic strokes that need to be managed. It has been demonstrated that AF patients with risks of ischemic stroke that are well-managed with NOACs are more likely to display thrombotic, proatherogenic, and proinflammatory factors than patients with ischemic stroke risks who do not have AF [9].

The CHA_2_DS_2_VASc score is designed for simplicity and for population-level risks [6]. More recently, the Calculator of Absolute Risk of Stroke (CARS) [10] and the modified CARS (mCARS) [11] were proposed for individual-level absolute risk assessments with and without OACs, but these has not been well-validated among Asian populations with AF. In contrast, the COOL-AF risk scores for SSE, bleeding, and death, which are based on multivariate weighted models, were derived and validated in an Asian AF population from Thailand [12].

The purposes of this study were (1) to validate the CARS and mCARS in a nationwide prospective COOL-AF registry of the population that was conducted in Thailand, and (2) to compare the CARS and mCARS models with the COOL-AF stroke risk model and the CHA_2_DS_2_VASc score for the prediction of SSE, i.e., for the prediction of occurrences of ischemic strokes or systemic emboli in patients after enrollment into the COOL-AF registry.

## 2. Methods

### 2.1. Study Population

The data concerning antithrombotic use and optimal INR levels in patients with non-valvular atrial fibrillation are from Thailand’s (COOL-AF) registry, which is a prospective AF registry from 27 hospitals in Thailand. The enrollment took place during 2014–2017. Patients with AF who were at least 18 years of age were enrolled. Patients with at least one of the following were excluded: (i) a mechanical heart valve, (ii) rheumatic valve disease, (iii) a life expectancy of less than 3 years, (iv) AF from a transient reversible cause, (v) inability to collect follow-up visit data, (vi) thrombocytopenia (a platelet count of less than 100,000/mm^3^), (vii) current participation in clinical trials, (viii) refused participation, (ix) ischemic stroke within the past 3 months, and (x) ischemic stroke during hospitalization. This study was approved by the Institutional Review Board of each study site. Each patient gave written informed consent before participation.

### 2.2. Study Protocol

The site investigators were encouraged to enroll consecutive patients. Site investigators explored data from medical records and interviewed patients for the information required to fill in the case record form. A central data management site verified that data were in the web system. A query process was performed as needed to ensure that the data were corrected. Study monitoring was performed at every study site to ensure that the study was performed according to the Good Clinical Practice (GCP) guidelines, to maintain data quality, and to ensure that the site investigators understood the study protocols and processes.

### 2.3. Data Collection

Site investigators were required to collect the following data: age; sex; weight; height; vital signs; symptoms; medical illnesses such as hypertension, type 2 diabetes (T2D), smoking, dyslipidemia, history of strokes, and coronary artery disease (CAD); and investigation data such as ECGs, echocardiograms, laboratory data, and medications for AF and other medical illnesses. Each item of every CHA_2_DS_2-_VASc score was recorded (C = congestive heart failure, 1 point; H = hypertension, 1 point; A = age > 75 years, 2 points; D = diabetes, 1 point; S = stroke, 2 points; V = vascular disease, 1 point; A = age 65–74, 1 point; and Sc = female sex category, 1 point) [6].

### 2.4. Outcomes

Follow-up data were required every 6 months until 3 years. The main outcome of this study was SSE. An ischemic stroke was defined as an acute focal neurological deficit lasting at least 24 h, or fewer than 24 h for TIAs. Imaging data from computerized tomography or magnetic resonance imaging were required to be uploaded to the web, but they could be negative findings. 

### 2.5. Prediction of the Risk of SSE 

In this study, the COOL-AF model developed for the prediction of SSE at 3 years was applied to the COOL-AF population using the variables in the final step of the multivariable analysis. We applied the CARS model for patients not using OACs [10] and the mCARS for those using OACs [11] in the COOL-AF population for the prediction of SSE. The COOL-AF model was created by the Cox proportional-hazard model with backward elimination and *p*-value < 0.05 as the stopping criteria. The following baseline variables were used: age (years), sex, body mass index (kg/m^2^), time after diagnosis of AF (years), AF types, symptomatic AF, history of heart failure, history of CAD, cardiac implantable electronic device (CIED), history of ischemic stroke/TIA, diabetes mellitus, hypertension, smoking, dyslipidemia, renal replacement therapy, dementia, history of systemic embolism, history of peripheral arterial disease, history of stent, history of CABG, history of alcohol abuse, history of bleeding, CKD, anemia, and anticoagulant use.

The CHA_2_DS_2_-VASc model was created using the Cox proportional-hazard model and the ENTER method from every variable used in the original CHA_2_DS_2_-VASc publication [6]. The CARS model was created using the Cox proportional-hazard model and the ENTER method of every variable used in the original publication of the CARS method [10]. Calculation of the absolute 3-year SSE risk, with death as a competing risk, was performed using cause-specific hazard regression models with a competing risk. With the input of the presence and date of SSE, presence and date of death, the program calculated the absolute 3-year SSE risk of each group with death as a competing risk, using the data to determine the absolute risk of SSE in patients who survived, estimate the chance of SSE if death had not occurred in those who did not survive, and come up with the final number of the absolute SSE risk treating death as competing risk. By doing this, the absolute risk of SSE is more accurate than without considering death as a competing risk [13].

The actual SSE risk was calculated for the whole population and for each CHA_2_DS_2-_VASc score. We calculated actual SSE risk and the SSE risk based on the COOL-AF model, the CARS, and the mCARS for each CHA_2_DS_2-_VASc score.

The probability of the occurrence of SSE at 3 years was calculated for each patient under the COOL-AF model, the CARS, the mCARS, and CHA_2_DS_2-_VASc using the equation derived using the Cox proportional hazards model for the COOL-AF population:

P_Outcome_ at 3 years = 1 − S_0_(t)^exp (Prognostic Index)^

where S_0_(t) is the average survival probability at time t (i.e., at 3 year), and the prognostic index is the sum of the products obtained from the multivariable analysis.

The models of the COOL-AF model, the CARS, and the mCARS for each CHA_2_DS_2-_VASc score are shown in Supplementary Table S1.

### 2.6. Statistical Analysis

Continuous data were described by mean and standard deviation (SD). Numbers (percentages) were used for categorical data. Comparisons of the continuous data were made using the student’s *t*-test for unpaired data, and comparison of categorical data were made by the chi-square test or the Fisher exact test. The incidence rate of clinical outcomes were described as the rate per 100 person-years at a 95% confidence interval (CI). The incidence rate of SSE was reported as incidence per 100 person-years. It was calculated from the number of SSE in each group divided by the sum of follow-up time of every patient in that group. The sum of the follow-up time was converted to 100 person-years before the calculation. The difference of incidence rates between the two groups were analyzed by the Poisson Regression (Incidence Rate Ratio).

#### Model Validation

Model applicability and risk of bias were assessed according to the Prediction model Risk of Bias ASsessment Tool (PROBAST) recommendations [14]. The calibration was tested using the calibration slope based on the observed and predicted hazards of SSE. A calibration slope of close to one shows a good agreement [15]. A receiver operating curve was used to determine Harrell’s C-statistics for the assessment of model discrimination. C-statistics vary from zero to one, and a value close to one indicates a good prediction model [16]. Receiving operating characteristics (ROC) curves and comparisons of ROCs were made.

All statistics were performed by the SPSS statistical software (SPSS, Inc., Chicago, IL, USA) and MedCalc Statistical Software version 12.5.0.0 (MedCalc Software bv, Ostend, Belgium). A *p*-value of less than 0.05 was considered to be of statistical significance.

## 3. Results

### 3.1. Study Population and Outcomes

**Figure 1:**
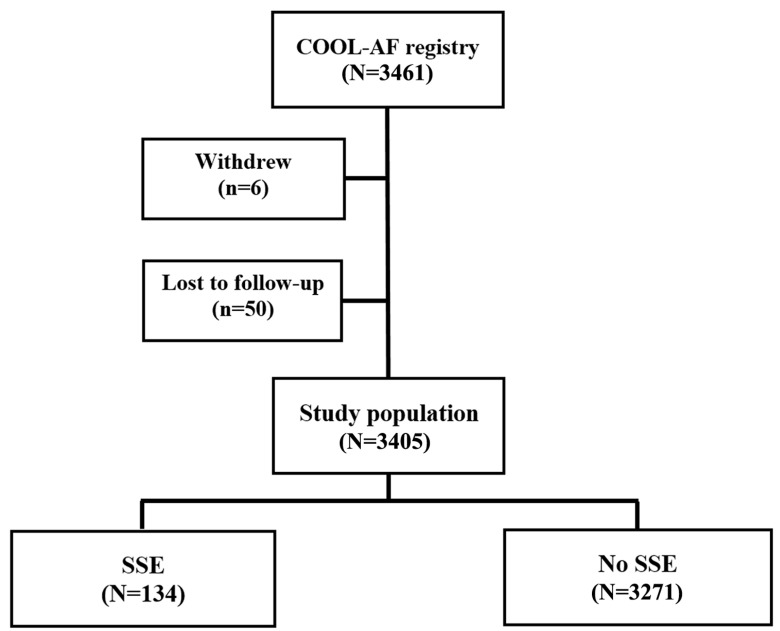
Flow diagram of the study population (SSE = ischemic stroke/systemic embolism).

We studied 3405 patients with a mean age of 67.8 ± 11.3 years, and 1424 (41.8%) were female. OACs were used by 2568 patients (75.4%), and warfarin was the major OAC prescribed (91.1% of OACs). The average follow-up duration was 31.8 ± 8.7, median (interquartile range) = 35.9 (34.8–36.00) months, or 9026.7 person-years. During the follow-ups, 134 patients had SSE (3.94%). The overall incidence rate of SSE was 1.51 (1.26–1.78) per 100 person-years. The baseline characteristics of the study population, as well as patients with and without SSE events, are shown in Table 1. Patients with SSE were older, more of them were female, and they had more cardiovascular risk factors or comorbid conditions than those without SSE. A flow diagram of the study population is shown in Figure 1.

### 3.2. SSE Risk According to the COOL-AF Model, CARS, mCARS, and CHA_2_DS_2-_VASc Scores

**Figure 2:**
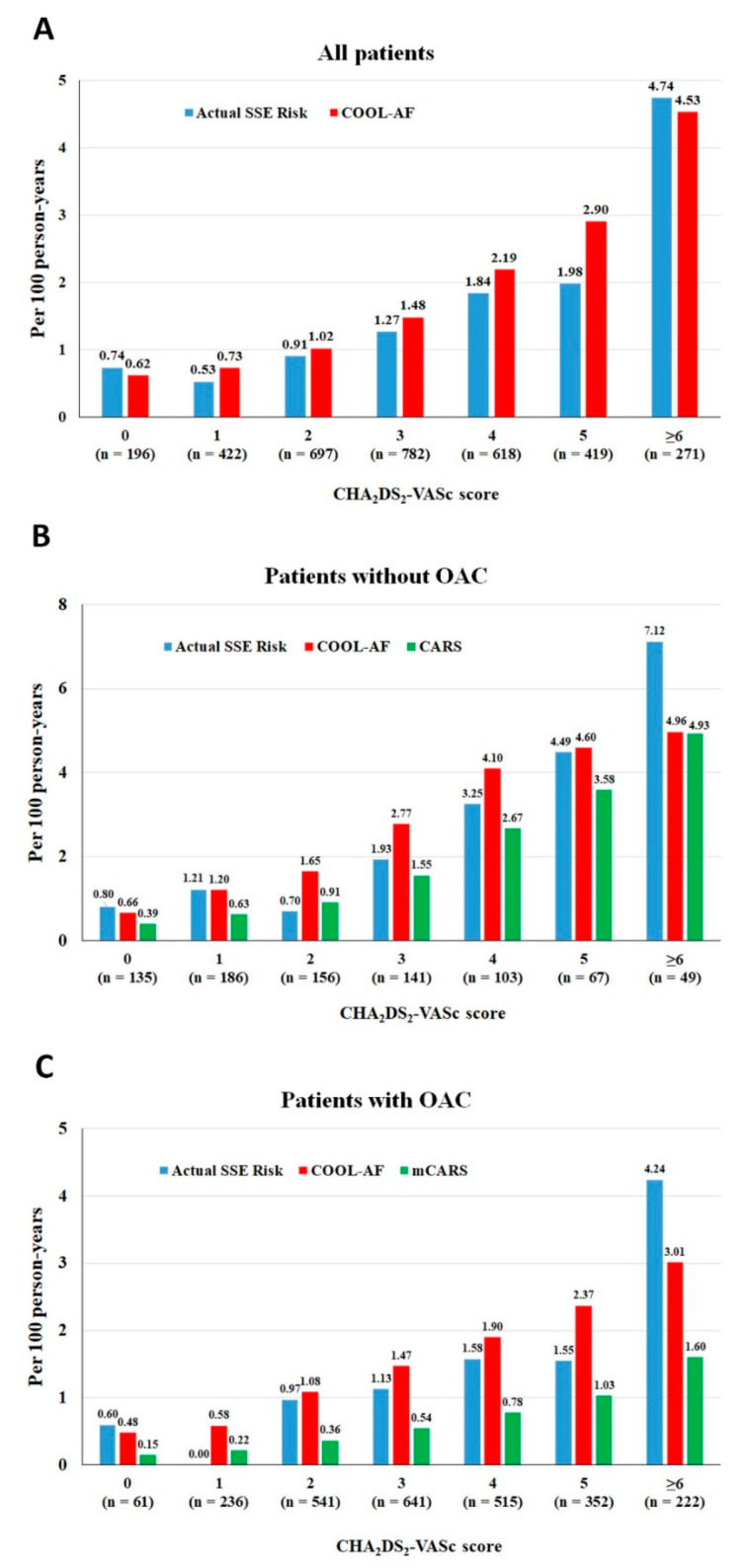
Bar graph of incidence rates of ischemic stroke/systemic embolism (SSE) of COOL-AF, CARS, and mCARS according to CHA_2_DS_2-_VASc scores for all patients (**A**), no-OAC patients (**B**), and OAC patients (**C**).

The overall incidence rates (and 95% CI) of SSE were 1.51 (1.26–1.78), 1.93 (1.39–2.60), and 1.37 (1.10–1.68) for all patients, no-OAC, and OAC patients, respectively. The absolute 3-year SSE risks with death as a competing risk were 4.12 (3.47–4.85), 5.15 (3.76–6.84), 3.79 (3.08–4.61) for all patients, no-OAC, and OAC patients, respectively. Table 2 shows the incidence rates of SSE from the COOL-AF model, the CARS, and the mCARS and the actual SSE risk for patients in each CHA_2_DS_2-_VASc score for the whole population as well as patients with and without OACs. The SSE risk according to the COOL-AF, CARS, and mCARS progressively increased as the CHA_2_DS_2-_VASc scores increased. Comparisons of the incidence rates of the COOL-AF, the CARS, and the mCARS according to the CHA_2_DS_2-_VASc scores are shown in Figure 2. The increased risks with the increase in the scores were consistent in both the OAC and the no-OAC group.

### 3.3. Comparisons of the COOL-AF Model, CARS, mCARS, and CHA_2_DS_2_-VASc Scores

**Figure 3:**
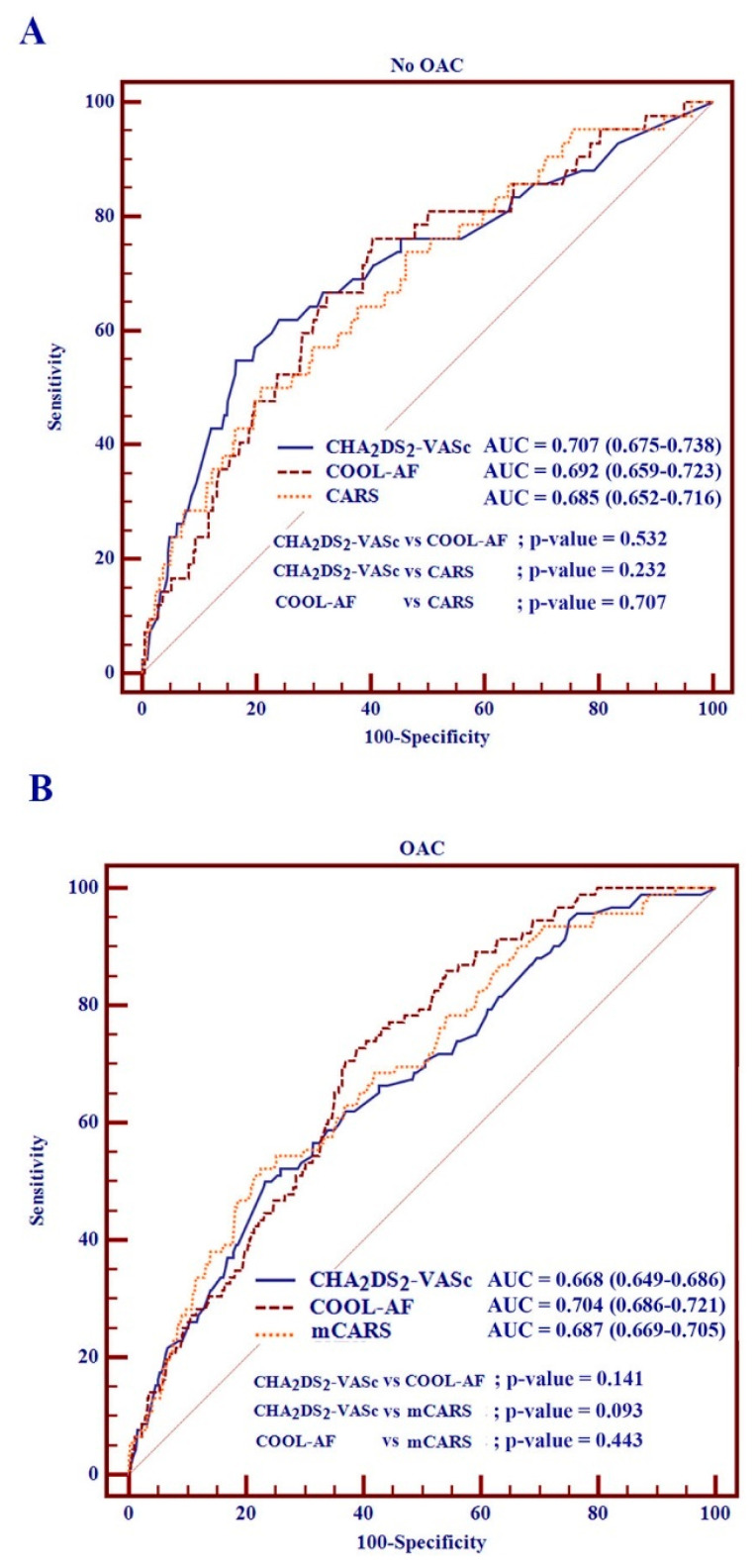
Comparisons of C-statistics of the COOL-AF, CARS, mCARS, and CHA_2_DS_2_-VASc, for the prediction of ischemic stroke/systemic embolism (SSE) for (**A**) patients without oral anticoagulants (OACs) and (**B**) patients using OACs.

Figure 3 shows C-statistics of (1) the COOL-AF model, the CARS, and the CHA_2_DS_2_-VASc scores for patients not using OACs and (2) the COOL-AF model, mCARS, and CHA_2_DS_2_-VASc scores for those using OACs. For the whole population, the COOL-AF had C-statistics of 0.697 (0.682–0.713), which was superior to the CHA_2_DS_2_-VASc [0.655 (0.639–0.671)]. For the no-OAC group, the CARS model can be used to predict SSE with a C-statistic of 0.685 (0.652–0.716), similar to CHA_2_DS_2_-VASc score [0.684 (0.651–0.7150] and COOL-AF model [0.692 (0.659–0.723)]. For the OAC group, the mCARS had a C-statistic of 0.687 (0.669–0.705), which was similar to the COOL-AF [0.704 (0.686–0.721)] but better than the CHA_2_DS_2_-VASc score [0.655 (0.637–0.674)] for the prediction of SSE.

**Figure 4:**
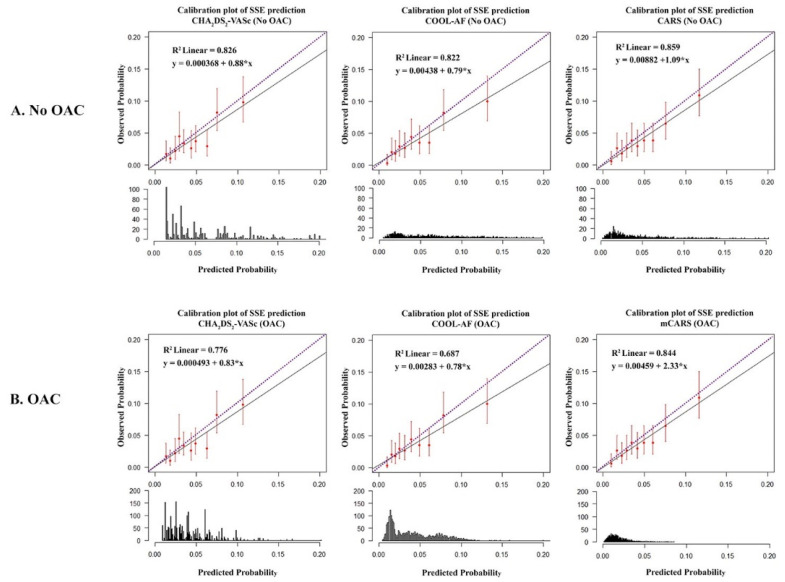
Calibration plots of the COOL-AF, CARS, mCARS, and CHA_2_DS_2_-VASc, for the prediction of ischemic stroke/systemic embolism (SSE) for (**A**) patients without oral anticoagulants (OAC) and (**B**) patients using OACs.

Calibration plots were performed to assess the predictive ability of each model against the observed (actual) risk on the Y-axis by dividing the predicted probability of each model into 10 equal groups. There were overestimation trends of the CHA_2_DS_2_-VASc, COOL-AF, and CARS models in the OAC-treated groups, as opposed to the observed risks in the no-OAC groups (Figure 4).

## 4. Discussion

The results of this prospective nationwide multicenter study in Thailand demonstrated that the CARS in patients who were not using OACs and the mCARS for patients using OACs can be used to predict SSE in Asian populations. The predictive ability of these models was equivalent to the COOL-AF prediction model and at least as good as the CHA_2_DS_2_-VASc score.

Prevention of ischemic strokes is an important management strategy for patients with AF [2,3,4] and can improve their life expectancy as well as their quality of life and social functioning. Identifying patients who should be treated with OACs is the key for ischemic stroke prevention. The CHA_2_DS_2_-VASc score was designed for simplicity and for population-level assessments [6], and it has been recommended in major practice guidelines [2,3,4]. Although the CHA_2_DS_2_-VASc score was developed using data from a Western population [6], it has been well-validated in Asian populations [12,17]. CHA_2_DS_2_-VASc scores help identify patients with a very low risk of ischemic stroke and who should not be treated with OACs, while all other patients with intermediate–high risks for ischemic strokes should be treated with OACs. Data in some Asian populations indicate that Asians might have more risk of ischemic strokes than non-Asians and, therefore, some Asian patients with CHA_2_DS_2_-VASc scores of 0 can still have an annual risk of 1.15% for ischemic strokes [18]. Hence, a modified CHA_2_DS_2_-VASc score can better identify Asian patients with a very low risk of ischemic strokes [17]. Since the Asian AF population may have different stroke and bleeding risk profiles, scoring systems based on non-Asian populations should be validated for their suitability for Asian AF patients [19,20]. Despite the recommendation of non-vitamin K antagonist oral anticoagulants (NOACs) as the preferred OAC over warfarin, data from many Asian countries showed that warfarin is the major OAC used by AF patients [21,22]. The proposed threshold to recommend OACs to AF patients was an absolute annual stroke risk of 1.7% for warfarin [7] and 0.9% for NOACs [23]. As the residual risk of ischemic strokes for AF patients remains even after OAC treatment, choice of OAC may be of importance. NOACs are at least as good as warfarin for ischemic stroke prevention. In addition, data from a previous study showed that AF patients with high prothrombotic factors such as high fibrinogen levels were well-protected from ischemic strokes with NOACs when compared to warfarin [24].

Even in AF patients who were on OACs, the calculation of SSE risk prediction still had benefits. It provided the residual risk of strokes. In patients whose residual risk remains high, physicians should focus more on patients’ compliance to takings the OACs, consider changing warfarin to NOACs, apply more risk-factor control strategies, and potentially communicate with patients regarding the residual risks. Regarding the possible benefit of NOACs over warfarin, we do not have direct data from this study. However, according to a previous study, the calculation of SSE risks derived from our study, would be beneficial for the decision not only to use anticoagulants, but also to choose NOACs over warfarin, since the threshold for the benefit over the risk of the NOACs for SSE was over 0.9% per year, but with warfarin, the threshold was over 1.7% per year [7].

The CARS model was developed by the national Danish AF registry by selecting only patients who were not on OACs [10]. The CARS was based on the calculation of the individual risk of SSE using a risk factor-based approach, which is different from using the average risk for a particular CHA_2_DS_2_-VASc score and may be able to improve risk estimates. Furthermore, the CARS model can assist in the communication of the stroke risks of individual patients and help in the shared decision-making of whether to initiate oral anticoagulation therapy. The results of our study suggest that the CARS method can be used to predict the absolute risk of SSE with C-statistics of 0.685 (0.652–0.716), results which were broadly similar to the CHA_2_DS_2_-VASc score and the COOL-AF model. The original CARS from the Danish registry had better C-statistics of 0.787 (95%CI 763–0.812), which was an improvement over the CHA_2_DS_2_-VASc score [0.733 (0.708–0.758)]. However, the number of patients not using OACs in our study was relatively small (*n* = 837 or 24.6%), which might explain the lower C-statistics of our study in comparison with the original CARS study from Denmark [10]. The results of our study supported the use of OACs, especially NOACs, since the actual risk of SSE was 1.21 (0.44–2.60) without OACs. The COOL-AF model was more accurate for SSE prediction, with a SSE risk of 1.2 (0.44–2.60) in patients with CHA_2_DS_2_-VASc of 1. The CARS underestimated the risk of SSE in this group [(0.63 (0.13–1.81)]. Therefore, caution should be exercised when applying the CARS model with Asian populations with CHA_2_DS_2_-VASc scores of 1.

The mCARS model was developed by Ding et al. [11] using the concept of an ischemic stroke reduction of 64% with patients using OACs compared to those not using OACs [25]. Therefore, they used the CARS model and calculated the ischemic stroke risk of patients using OACs by multiplying the results by 0.36. There are some limitations of using the mCARS model, such as that (1) the mCARS uses calculations from the CARS but was not developed with data from a no-OAC group, and (2) the calculation of the mCARS using the 64% risk reduction for patients using OACs was derived from historical studies based on warfarin treatment, which may not be applicable in the NOAC era. The results of our study demonstrated that the mCARS had a C-statistic of 0.687 (0.669–0.705), which was similar to the COOL-AF [0.704 (0.686–0.721)] and better than the CHA_2_DS_2_-VASc score [0.655 (0.637–0.674)] for the prediction of SSE in OAC-treated patients. Our results suggest that the mCARS model can probably be used in Asian AF populations. Among patients with CHA_2_DS_2_-VASc = 0 who received OACs, the actual SSE risk was 0.6 (0.01–3.28) per 100 person-years, whereas the incidence rate was 0.48 (0.01–2.64) according to the COOL-AF model and 0.15 (0.01–0.80) according to the mCARS. This result indicates that the mCARS method underestimated the risk of SSE in Asian populations with CHA_2_DS_2_-VASc = 0 who received OACs. Previous studies from Taiwan [17] and Hong Kong [26] have demonstrated that the risk of SSE of Asian populations with CHA_2_DS_2_-VASc = 0 are higher than indicated by data from non-Asian populations.

The purpose of this study was to determine whether the CARS and mCARS models, which were developed from the results of non-Asian populations, can be applied to Asian populations. The results of our study indicated that the CARS and mCARS models can be used for Asian populations with similar predictive values to the COOL-AF model and the CHA2DS2-VASc model. While CHA2DS2-VASc is a scoring-based system for risk prediction, the COOL-AF, CARS, and mCARS models provide absolute risk predictions. Despite the fact that CHA2DS2-VASc scores may be easier to use, models with absolute risk prediction are probably more accurate and may be more precise for communication with patients.

Among 2787 patients with CHA_2_DS_2_-VASc scores above 2 points, 1984 (71.2%) were on OACs alone, 287 (10.3%) were on OACs plus antiplatelet medications, 415 (14.9%) were on antiplatelet medications alone, and 101 (3.6%) were taking no antithrombotic drugs. Overall, 2271 (81.5%) patients were on OACs. The rate of OAC use among patients with CHA_2_DS_2_-VASc scores ≥ 2 in our study was greater than the reported rate of the Asian population in the GARFIELD registry and similar to the rate of non-Asian populations. Despite the fact that patients with CHA_2_DS_2_-VASc scores ≥ 2 should be on OACs, there are many factors that influence decisions concerning OAC treatment, including fear of bleeding or a history of bleeding, especially since the main OAC used in our study was warfarin.

Left atrial appendage occluders (LAAOs) are an alternative option for the prevention of strokes in AF patients, especially for those who cannot be on OACs. Recent data has shown good long-term results with acceptable complication rates, even in elderly populations [27]. The procedure can be performed by endocardial or epicardial approaches with a similar mid-term follow-up [28]. Results from a nationwide study in the United States indicated that endocardial approach seemed to have a lower complication rate than the epicardial approach [29]. The new discoveries in LAAOs might further elevate the need for new and much more precise risk scores, such as altered coagulation factors and fibrinolytic activity in the left atrial appendage [30], metabolic effects of the left atrial appendage exclusion [31], and the systemic hemostasis that might be affected after left atrial appendage ligations [32].

### Limitations

There are some limitations of this study. First, whether or not OACs were used of may have changed with time. Therefore, the choice to use or not use OACs at the baseline may not represent the OAC status for the whole period of the study. Second, warfarin was the major OAC used in our study. Therefore, the results may not be fully generalized to other populations which mainly used NOACs. Third, 56 patients (1.6%) were lost to follow-up or withdrew from the study. Fourth, the adjustment of covariates was performed by the Cox proportional-hazard model during the creation of the COOL-AF model. The factors that are not independent predictors for SSE were eliminated during the Cox proportional-hazard model. However, the medications that were in the model were OACs. We did not include treatment of hypertension or dyslipidemia in the models. We believed that patients who received these medications may be at a higher risk and may make it difficult to interpret the model.

## 5. Conclusions

The calculation of the individual absolute risks using the CARS and mCARS models, which are based on using a risk factor-based approach, can predict SSE in an Asian population. Small differences were evident compared to the COOL-AF and CHA_2_DS_2_-VASc scores.

## Figures and Tables

**Table 1:** Baseline Characteristics of the study population.

Characteristics	SSE (*n* = 134)	No SSE (*n* = 3271)	*p*-Value
Age (years)	73.5 ± 9.7	67.6 ± 11.3	<0.001 *
Female gender	73 (54.5%)	1351 (41.3%)	0.002 *
BMI (kg/m^2^)	24.6 ± 4.8	25.2 ± 4.7	0.172
Time after diagnosis of AF (years)	3.8 ± 4.9	3.4 ± 4.3	0.327
Atrial fibrillation			0.504
Paroxysmal	39 (29.1%)	1109 (33.9%)	
Persistent	28 (20.9%)	617 (18.9%)	
Permanent	67 (50.0%)	1545 (47.2%)	
Symptomatic AF	103 (76.9%)	2517 (76.9%)	0.982
History of heart failure	43 (32.1%)	870 (26.6%)	0.160
History of CAD	26 (19.4%)	521 (15.9%)	0.283
CIED	14 (10.4%)	327 (10.0%)	0.865
History of ischemic stroke/TIA	32 (23.9%)	560 (17.1%)	0.043 *
Diabetes mellitus	45 (33.6%)	794 (24.3%)	0.014 *
Hypertension	109 (81.3%)	2221 (67.9%)	0.001 *
Smoking	25 (18.7%)	653 (20.0%)	0.710
Dyslipidemia	77 (57.5%)	1840 (56.3%)	0.782
Renal replacement therapy	4 (3.0%)	36 (1.1%)	0.070
Dementia	1 (0.7%)	28 (0.9%)	0.892
Systemic embolism	3 (2.2%)	22 (0.7%)	0.073
History of peripheral vascular disease	6 (4.5%)	38 (1.2%)	0.007 *
History of stent use	9 (6.7%)	244 (7.5%)	0.748
History of CABG	5 (3.7%)	60 (1.8%)	0.111
History of alcohol abuse,	5 (3.7%)	135 (4.1%)	0.821
History of bleeding	19 (14.2%)	305 (9.3%)	0.060
CKD	101 (75.4%)	1655 (50.6%)	<0.001 *
Anemia	76 (56.7%)	1217 (37.2%)	<0.001 *
Anticoagulant	92 (68.7%)	2476 (75.4%)	0.064
Warfarin	87 (64.9%)	2253 (68.9%)	0.333
NOACs	5 (3.7%)	223 (6.8%)	0.161

Data are presented as mean ± standard deviation or number and percentage; * *p*-value < 0.05 indicates statistical significance. Abbreviations: SE—ischemic stroke/systemic embolism, AF—atrial fibrillation; BMI—body mass index, CAD—coronary artery disease, CIED—cardiac implantable electronic device; TIA—transient ischemic attack, CKD—chronic kidney disease, NOACs—non-vitamin K antagonist oral anticoagulants.

**Table 2:** Incidence rates of ischemic stroke/systemic embolism (SSE) of the COOL-AF, the CARS, the mCARS according to the CHA2DS2-VASc scores for all patients (*n* = 3405), OAC (*n* = 2568) and no-OAC (*n* = 837) patients.

CHA_2_DS_2_-VASc	*n*	COOL-AF (All)	COOL-AF (No OAC)	COOL-AF (OAC)	CARS (No OAC)	mCARS (OAC)	Actual SSE Risk (95% CI)	Actual SSE Risk (95% CI) (No OAC)	Actual SSE Risk (95% CI) (OAC)
0	196	0.62 (0.11–1.59)	0.66 (0.06–1.90)	0.48 (0.01–2.64)	0.39 (0.01–2.19)	0.15 (0.01–0.80)	0.74 (0.20–1.86)	0.8 (0.16–2.30)	0.6 (0.01–3.28)
1	422	0.73 (0.30–1.36)	1.2 (0.44–2.60)	0.58 (0.16–1.48)	0.63 (0.13–1.81)	0.22 (0.01–1.17)	0.53 (0.19–1.13)	1.21 (0.44–2.60)	0 (0.00–0.00)
2	697	1.02 (0.60–1.56)	1.65 (0.64–3.30)	1.08 (0.61–1.73)	0.91 (0.24–2.30)	0.36 (0.12–0.83)	0.91 (0.52–1.43)	0.7 (0.14–2.01)	0.97 (0.52–1.60)
3	782	1.48 (0.97–2.06)	2.77 (1.30–4.98)	1.47 (0.94–2.15)	1.55 (0.56–3.33)	0.54 (0.25–1.03)	1.27 (0.82–1.83)	1.93 (0.76–3.91)	1.13 (0.67–1.73)
4	618	2.19 (1.52–3.04)	4.1 (1.92–7.36)	1.9 (1.21–2.76)	2.67 (1.06–5.42)	0.78 (0.37–1.41)	1.84 (1.21–2.60)	3.25 (1.38–6.31)	1.58 (0.96–2.37)
5	419	2.9 (1.96–4.09)	4.6 (1.78–9.13)	2.37 (1.44–3.57)	3.58 (1.30–7.68)	1.03 (0.46–1.92)	1.98 (1.21–2.98)	4.49 (1.78–9.13)	1.55 (0.83–2.56)
≥6	271	4.53 (3.03–6.51)	4.96 (1.61–11.55)	3.01 (1.71–4.86)	4.93 (1.61–11.55)	1.60 (0.69–3.15)	4.74 (3.22–6.72)	7.12 (3.08–14.07)	4.24 (2.69–6.37)
All	3405	1.76 (1.47–2.02)	2.22 (1.60–2.88)	1.61 (1.29–1.90)	1.46 (0.99–2.04)	0.66 (0.47–0.87)	1.51 (1.26–1.78)	1.93 (1.37–2.57)	1.37 (1.09–1.66)

## Data Availability

The dataset that was used to support the results and conclusion of this study are included within the manuscript. The additional data are available from the corresponding author upon reasonable request.

## Supplementary Materials

The following supporting information can be downloaded at: https://www.mdpi.com/article/10.3390/jcm12072449/s1, Table S1: Predictive models for ischemic stroke/systemic embolism (SSE) of the COOL-AF model for all patients, patients using oral anticoagulants (OACs), and those with no OACs.
