# Supplementary material for: Predicting the Absolute Risk of Ischemic Stroke in Asian Patients with Atrial Fibrillation: Comparing the COOL-AF Risk Score with CARS/mCARS Models for Absolute Risk and the CHA2DS2-VASc Score
Source: J Clin Med. 2023 Mar 23;12(7):2449. doi: 10.3390/jcm12072449 (PMC10095200; doi:10.3390/jcm12072449)
Supplement: Supplementary file 1 [file jcm-12-02449-s001.zip › jcm-2230942-supplementary.pdf]

Supplementary Materials

Table S1. Predictive models for ischemic stroke/systemic embolism (SSE) of the COOL-AF model for all patients, patients using oral anticoagulants (OACs), and those with no OACs.

**All Patients**
Predictive model of CHA_2DS_2-VASc
$P_{SSE\ at\ 3\ years} = 1 - 0.98502744^{exp\ (Prognostic\ Index)}$ where Prognostic Index = 0.213823* History of heart failure + 0.390721* Hypertension + 1.051459* Age ≥ 75 years old + 0.326273* Diabetes mellitus + 0.373963* History of ischemic stroke/TIA + 0.209704* Vascular disease + 0.635813* Age 65-74 years old + 0.418353* Female gender
Predictive model of COOL-AF
$P_{SSE\ at\ 3\ years} = 1 - 0.99757575^{exp\ (Prognostic\ Index)}$ where Prognostic Index = 0.036315* age (years) + 0.421472* Female gender + 0.430014* History of ischemic stroke/TIA + 0.451208* Diabetes mellitus + 0.951090* PAD + 0.608073* CKD - 0.621781* OAC
**No-OAC patients**
Predictive model of CHA_2DS_2-VASc
$P_{SSE\ at\ 3\ years} = 1 - 0.98502744^{exp\ (Prognostic\ Index)}$ where Prognostic Index = 0.031426* History of heart failure + 0.773855* Hypertension + 0.908745* Age ≥ 75 years old + 0.558121* Diabetes mellitus + 0.288945* History of ischemic stroke/TIA + 0.080391* Vascular disease + 0.579825* Age 65–74 years old + 0.425016* Female gender
Predictive model of COOL-AF
$P_{SSE\ at\ 3\ years} = 1 - 0.99809532^{exp\ (Prognostic\ Index)}$ where Prognostic Index = 0.032248* age (years) + 1.393322* PAD + 0.708500* CKD + 0.525858* Anemia
Predictive model of CARS
$P_{SSE\ at\ 3\ years} = 1 - 0.99931304^{exp\ (Prognostic\ Index)}$ where Prognostic Index = 0.049933* age (years) +0.373918* Female gender + 0.240865* History of heart failure + 0.330411* History of ischemic stroke/TIA + 0.339338* Diabetes mellitus + 0.326092* Hypertension + 0.186039* PAD or MI
**OAC Patients**
Predictive model of CHA_2DS_2-VASc
$P_{SSE\ at\ 3\ years} = 1 - 0.99079605^{exp\ (Prognostic\ Index)}$ where Prognostic Index = 0.305608* History of heart failure + 0.283172* Hypertension + 1.207893* Age ≥75 years old + 0.256090* Diabetes mellitus+ 0.504818* History of ischemic stroke/TIA + 0.214460* Vascular disease + 0.742674* Age 65-74 years old + 0.420445* Female gender
Predictive model of COOL-AF
$P_{SSE\ at\ 3\ years} = 1 - 0.99798039^{exp\ (Prognostic\ Index)}$ where Prognostic Index = 0.039977* age (years) + 0.940397* Hypertension
Predictive model of mCARS
$P_{SSE\ at\ 3\ years} = P\ (CARS)_{SSE\ at\ 3\ years} * 0.36$

See abbreviation list from Table 1.
